# Public School Instruction in Oral Hygiene

**Published:** 1910-02-15

**Authors:** Paul G. White

**Affiliations:** Boston, Mass.


### PUBLIC SCHOOL INSTRUCTION IN ORAL HYGIENE.

DR. PAUL G. WHITE, BOSTON, MASS.

Much time has been spent in a search for that something that will increase the length of man's life, improve his health and, incidentally, add to his happiness. For hundreds of years search has been made and numerous remedies declared quite sure to succeed if faithfully used, but all of these have been short-lived disappointments. It may be that we will yet find such a remedy or panacea, but we rather incline to the belief that the prolongation of life will follow right living, correct diet and the observance of hgyienic laws, and prophylactic measures that will keep us free from infection and contagion.

While there has been a great deal of scientific advancement more recently along certain lines of investigation which have been progressive for the healthfulness of people in general, still we can see abundant evidence of people, perhaps a majority of whom are women and children, whose physical condition and apparent healthfulness are not what we might expect.

We know that people suffering from organic and functional nervous and mental affections are becoming rather alarmingly common, and that people suffering from various bodily afflictions and physical defects, who are unable to earn a living and are a charge upon benevolence of some kind, are becoming more numerous.

That our methods of living at the present time may have something to do in the bringing about of these conditions there is not much doubt. It is evident that the strenuous life in our modern public school system in the past, and even at the present time is not yet so perfect, but that it still affords anually a small army of broken-in-health, nervously afflicted, deformed, pitiable recruits.

To-day conditions have somewhat improved, but there is still great need for further improvements in the development of the bodies of the school boy and girl, for it must not be forgotten that the scholar consists of a body as well as a brain, and that the physical constitution must suffer if the body is neglected in order that the brain may be too rapidly worked.

Our young people are anxious to "learn." They crowd our colleges and are in turn crowded full of all the accumulated knowledge of the ages, while little, if any, attention is given to what they eat, where they sleep, or regarding what care they give their bodies. They are finally graduated with honors and some of them never make any use of the knowledge gained, for of what use is it to a semi-invalid who knows nothing of the care necessary to keep the thinking and working machinery in good condition? They are taught that everything in this world moves along according to law *excepting our own bodies;* here they are encouraged to believe that one may continually violate natural laws and when illness comes a healer may be employed who will pray or drug them back to health. To one who has gotten his eyes thoroughly opened, this appears extremely absurd, but isn't it just what our young people are being taught? Drugs by one healer or prayer by the other will be so powerful that the very laws of life and death may be violated with impunity.

If it is true that the public schools have served as an incubator for the development and the dissemination of more disease than any other single cause known, then is it not the duty of the school to arouse its pupils to intelligent thought on the importance of better modes of life? Surely to compel a child to attend school and subject it to infection that may result in sickness or death, if conditions making this possible are known to those in authority, is nothing short of an unjustifiable crime.

If every individual practiced rules necessary to escape from infectious disease, public hygiene—chiefly concerned with preventing the spread of infection—might go out of business; thus personal hygiene becomes the basis of public hygiene. For the man who lives on his own estate, conforming to the rules of hygiene, public hygiene need not exist, but if he leaves his estate and travels into society public hygiene steps in. Public hygiene says, "What you do for yourself at home, we do for you in public."

Hygienists should especially consider school children under three heads, 1st, age; 2nd, surroundings and close contact; 3rd, special conditions, connected with education.

The age period may be said to exist between the 5th and 15th years. It is here that we find the highest mortality rate and this is due almost wholly to communicable, hence preventable, diseases. As this period is the largest determining factor in the future efficiency of the child's mind, so is it the time during which the future efficiency of the body is decided for the good or bad. Our state laws declare that whatever else is done with a child's time in school he shall be taught hygiene and physiology. The cursory and indifferent attention paid to hygiene can be evidenced by almost any school curriculum. The garbled and inferior text books used in many states are devoted largely to the evil effects of alcohol and tobacco, and there is not, to my knowledge, a book used in our public schools to-day that contains a chapter on oral hygiene, which should be the longest and most important in the whole book, and we come to the conclusion that the state has failed lamentably on this fundamental topic of education and that hygiene, as taught in the public schools, is a sinister practical joke. The remedies are self evident and can be briefly stated: 1st to teach real hygiene in schools by adequate teachers and text books; 2nd, to sweep our present farce out of the schools and consign it to the junk heap where it belongs.

If medical inspection in the public schools has been inaugurated to prevent the spread of disease among children attending the schools, then it seems to me that such prevention of diseases ought not to be wholly confined to the prevention of infectious diseases only. Let such inspections go a little deeper. Let the medical inspectors remove the predisposing causes of the maladies children are prone to contract at school.

A brief consideration of the predisposing causes will not be out of place.

As the oral cavity has been fittingly termed the "Gateway of Life," so may oral hygiene be correspondingly styled the "Gateway to Health," or the key to all personal and domestic hygiene. As personal and domestic hygiene is closely related to sanitary science, this same key may open the way to a larger and broader application of state and municipal hygiene. Oral hygiene may be defined as that sub-division of gneral hygiene treating of the care and proper use of the mouth and teeth. As the healthy body depends upon the air it breathes and the food it assimilates and as both of these are directly affected by the conditions in the oral cavity it is not an exaggeration to say that three-fourths of the ills of mankind will be banished as soon as the mouth and teeth receive the care and attention they require.

The important question which arises here is, "How is this care and attention to be brough about?" The answer, "By educating the public," and then again and unanswered question, "How?"

As a worthy example set by a large city is readily followed by smaller cities and towns, let us consider what has been done in our own city of Boston towards establishing oral hygiene in the schools.

In the first place, our Massachusetts Medical Inspection law calls for the physical examination of every school child for any disability or defect, tending to prevent his receiving the full benefit of his school course. If we consider dentistry a branch of medicine, and who will say it is not, I submit to you that the laws are ineffective.

Now, because of the ineffectiveness, I took the matter of oral hygiene before the Boston School Board and the Boston Schoolmasters' Association in the spring and fall of 1907, through the influence of Hon. J. F. Fitzgerald, then mayor of Boston. Upon suggestion of the Superintendent of Schools, I read a paper on "The Necessity of School Instruction in Oral Hygiene" before some three hundred schoolmasters. This paper created considerable enthusiasm, and the masters made many requests for lectures on the care of the mouth and teeth in the schools of Boston, which were given and proved to be a great success. In April a paper entitled "More About School Instruction in Oral Hygiene," widely distributed among the masters, added greatly to the interest already shown. This was followed by a petition to the School Board asking for the establishment of a course of Oral Hygiene in the schools. This petition was referred by the School Board to the Board of

Superintendents for consideration and report, and at a meeting of the School Committee on February 20, 1908, they reported as follows:

"The board heartily appreciates the desirability of inculcating habits of dental cleanliness among school children, and that much attention is given to the subject by many teachers. The Board of Superintendents believe that this is a matter which should properly engage the attention of the Department of Physical Training and Athletics, and recommends that it be referred to that department to consider means and methods for making instruction along these lines more effective.

The communication in which this report was embodied was placed on file and the recommendations of the Board of Superintendents were adopted by the School Committee.

By request of the Director of Physical Training at a meeting shortly after February 20th he informed me that if I could get the endorsement of the Metropolitan District of the Massachusetts Dental Society he could practically assure me that some kind of a course would be established during the coming year. Upon presentation of this proposition to the Metropolitan District it was referred to the newly established Dental Hygiene Council of Massachusetts, that is, it took an unnecessary trip out of the hands of the Metropolitan Society and that is where it is to-day. Now, gentlemen, I ask what you are going to do about it?

They say that the far reaching relations of dental health or disease to health or disease in general are as yet very little appreciated. How, then, may they be appreciated? Will dental examinations alone bring this about? Perhaps, —but with the best result,—No! Cities and towns are not as a general rule in condition financially to undertake the proper care of school children's teeth and, even if they were, it is most uncertain whether this would be the best way out of the difficulty. Then, while lectures are productive of much good they only reach a comparatively small part of the population, whereas a course in oral hygiene would permanently affect the entire generation. The want of a suitable text book, however, makes us rather timid about approaching school authorities and it seems to me that this can only be rectified in the following manner:

Perhaps the reading of two letters will illustrate what I wish to say. As a member of the Oral Hygiene Committee of the National Dental Association I have written to a number of authors of the various text books on Physiology and Hygiene used in the public schools of this country the following letters:

"Dear Sir:

As there is at present time no hygiene or physiology used in our schools or colleges which contains a chapter on oral hygiene, I am writing to ask if you would be willing to have incorporated in your book a chapter on oral hygiene compiled and copyrighted by the National Dental Association. We are endeavoring to have oral hygiene taught in every public school in the Union and this seems to be the only way to obtain a uniform course of instruction. I should be glad if you would make any suggestions in regard to the same.

Sincerely yours,

(Signed) PAUL GARDINER WHITE."

A reply from Dr. Seneca Egbert, one of the leading hygienists of this country, reads in part:

"Shortly after receiving your letter I consulted my publisher as to the advisability of your proposal and found that he was quite willing to consider it seriously. He suggested that it might be well to ask you to send on the manuscript of the proposed chapter, so that we might judge not only of the subject matter but also as to how much space it would take. As I understand it, there is not much likelihood of there being another edition of my book published this year, but my publisher informs me that your chapter might go in as an insert in copies not yet bound just before the index together with an explanatory paragraph or two.

Yours sincerely,

(Signed) SENECA EGBERT."

Now do not these letters bring out the best way to introduce oral hygiene all over this country in the shortest possible time without objection by School Boards, Boards of Education, Legislature, etc., on account of expense?

The suggestion of Dr. Corley, of the National Committee, that the State Association create standing committees to act as sub-committees to the National Committee with such changes and modifications as may be thought best to meet the needs of their special fields, and that they submit an annual report to the National Committee is, I believe, a very necessary step to carry on this national campaign. If this co-operation cannot be secured, then of what real use is our National Dental Association.

As a member of the Oral Hygiene Committee of the National Dental Association it is my duty to present and urge the following out of our plans for the coming year.

First, we wish the president of every state society in the Union to appoint a committee on oral hygiene to work in connection with the National Committee, and to have, through them, every sub or independent organization in the States elect or appoint committees to work in connection with us. If this can be done we will have every organization that is working along the oral hygiene line report to us directly upon what they are accomplishing, and when anything new that is worthy of consideration comes from any source whatsoever it will be compiled along with other good suggestions, printed and mailed to the other committees at work throughout the country as well as to school boards, chambers of commerce, boards of health, charity institutions and every other organization which may be brought into play to advance the interests of this work.

This will give us the benefit of the best minds throughout the entire country, and will send a wave over the land which will do far more good in one year than could be accomplished in years by each individual organization working separately.

The great need of popularizing oral hygiene may be shown by the following report of the United States Census which shows a population of 80,000,000 people constantly increasing. Statistics show that less than ten per cent. of these ever patronize the dentist. From these figures we may say that almost 70,000,000 people need information on oral hygiene. If every person in the United States went to the dentist each dentist would have 2,500 patients on his list. As only ten per cent. go it averages 250. And people say there are too many dentists! It is said that all the dentists in this country, working eight hours a day the rest of their lives, could not begin to do the work for the people of Greater New York needing dental services.

Individually and collectively we can do much in diffusing dental knowledge.

One cannot be brought face to face with an earnest company of school teachers, numbering 1,500 or 2,000 men and women, such as recently assembled, without having some comprehension of the relation which such a body of workers sustains to the future of our city and state, and without feeling how large a place they should occupy in the respect and interest of all classes of our urban population.

This is a matter in which we hope not only the members of our organizations, but also every village improvement society, grange and women's club will be interested. It is an especially appropriate subject for women to take hold of, inasmuch as the whole effectiveness of the movement will depend upon the degree of interest which children's mothers take.

If women at their clubs would spend more time on the subject of the education of their own children, rather than studying the old masters, French and German literature, the reading of trashy books, and playing bridge, the conditions surrounding the children of this country, which would tend to make the sound in body and mind, would be all that we desire.

A movement is on foot in Boston at the present time, and it includes the whole state, to give us a newer, better, busier, cleaner, and more healthful city in 1915. Can this be done effectually without the introduction of oral hygiene into the public schools?

Let us raise our banners to the public gaze inscribed, "Oral Hygiene in 1915."

### DISCUSSION.

DR. BELYEA—I am going to ask you to consider the clinic at the Harvard Dental School. In pursuance of a plan formulated last year a committee called upon the dental schools to form a clinic. As you know by the report from the committee, this has been done in that there is a very good clinic at the Harvard Dental School and it works in conjunction with and under the supervision of this organization that we call the Metropolitan Dental Hospital Association. Volunteers to the number of something like fifty have been enrolled. New ones are constantly available and we hope to have a good clinic, and improve it when the school is moved up to the Huntington avenue building. I will answer any question that may be asked about it.

*Question.* Do you consider it a practicable thing to conduct a dental clinic as you would conduct a private practice, particularly in respect to the teeth? There are a large number of children having poor teeth and without private dentists. Is it practicable to treat these the same as in a private office?

*Answer.* It depends upon the number of schools and the number of children. If too much work presents itself you can do only a little. In such a clinic you will have to extract and do the best you can. You will have both work and instruction in trying to improve the little ones' teeth. At the Harvard clinic they are making a very small charge to all hands except when they cannot pay even this. In such cases the charitable organizations pay the fee. They are sent to us by some organization. At present this work is doing individual dentists as much good as it is the children.

*Question.* What charges are made and how many patients can you take each afternoon?

*Answer.* At present we have the use of the Infirmary only two afternoons, from 2 P. M. to 4.30 or 5 P. M. I have earned as high as $1.65 in one afternoon. Fillings cost from 10 to 25 cents, extractions 10 cents, etc. We want to teach them to co-operate by paying a little. As to the number of patients, I have had as high as five for treatments, extractions, etc. Usually two or three are all I can do as these patients have each a great deal of work to be done. We try to get the mouth into some sort of order.

*Question.* How do the ages average?

*Answer.* From five or six to twelve years or sometimes older. The first patient I had was a girl who was being sent to one of the Boston schools.

*Question.* Do these patients come from the poorer classes? Do some children come whose parents might pay for them?

*Answer.* This has not yet occurred, as they are indorsed by some organization.

*Question.* Do you find you have considerable extraction to do?

*Answer.* Practically little. Mostly saving, and treatments of temporary teeth. In this connection I would state that the Central District is about to open a clinic in Worcester early in July and has secured splendid quarters.

DR. BOARDMAN.—There has been a clinic of another character formed in Brookline through the initiation of the superintendent of schools. The town is paying for the dental work of the poorer children.

*Question.* Is there a similar clinic elsewhere?

*Answer.* I have a letter from Tufts College stating that they are to have a similar clinic. There ought to be a free interchange of the graduates of both schools, neither schools nor dentists making any difference, all co-operating.

*Question.* Is there any work now being done at the Tufts School?

*Answer.* Yes, but the work at present is done entirely by students. They have a large number and are doing excellent work. In the clinics the children are instructed that they must have their teeth clean before any work is done.

DR. POND of Franklin, Mass.—In our village we do not have quite the same proposition you city dentists have. There is occasionally a chance to do some missionary work among the children. There occasionally come to me some cases for which I could not get any fee for my work. We do not have a large class of poor children to work for and I think in the town of Franklin practically all of the people have a good idea of taking care of their own teeth, so that we have less charitable work to do.

A short time ago our local dentists, all of whom are members of the Massachusetts Dental Society and among whom a cordial feeling exists, held a well attended meeting before the Franklin Business Men's Association, at which talks were delivered by Dr. Edward Otis on "Oral Hygiene in Relation to the Great White Plague, and General Diseases," and by Dr. W. H. Potter on "Care of the Teeth, with Especial Reference to Prevention." These talks gave the public a better idea of the importance of our profession. In the fall we hope to present the matter at a meeting before the Alden Club, a ladies' organization.

DR. BELYEA—Practically all dentists have many calls for charitable work and practically every dentist is doing lots of this work. But the public does not know that we take any interest in such matters. We should not hide our light under a bushel. The members of the Massachusetts Dental Association in Boston number about three hundred and fifty and they are about all we can depend upon to help in this work, since if a man does not take enough interest to join the Society he will rarely do anything else.

Dr. Hosley, Springfield—I have been very glad to hear these papers. Very sensible ideas have been brought out for our use. We should all gather a certain amount of inspiration from these papers. I was especially interested in the little experiences that Dr. Lindstrom brought out in his papers. They are of a character educational to the profession. It may be a question as to what are the best means to the end. I think that the education of the children in this matter has perhaps been best brought out in the work which the German government is doing. It was discovered in the Army Field Day Exercises that the men were not able to do the work which the regulations called for. Investigation developed the fact that most of the soldiers were unable to masticate their food. The medical report was that the present conditions should be remedied, and it seemed best that money should be expended upon the common soldier.

In Germany the government is also taking up the examination of the school children's teeth and in many of the States it is compulsory and many municipalities are obliged to pay for keeping the mouths of the school children in a sanitary condition. If a government considers this important how much more does this deserve attention from each one of us as individuals? Really, there are dental conditions in civic life which are of great importance in respect to the spreading contagion, and the condition of the mouth has an important bearing upon this subject. It seems to me that a propaganda on this subject might bring the good results and should be begun by the dental profession.

I think Dr. White's paper a very good one and I am rather surprised that this work has not been done before. The individual dentist has a good chance to help along this good work at his chair. I think Dr. Lindstrom and his clinic will accomplish splendid results.

We have had a little experience in Springfield. It seems that our Board of Health formed an opinion that no good could be accomplished in a free clinic, and we disagreed. The educational effect of the dental hospital is worth all the effort. I think the profession will find the difficulty will be to find sufficient help to handle the tremendous undertaking. And I find the individual dentists are doing a great amount of charitable work. The medical profession says we do no charitable work. I complain that we have not received credit. Such papers as we have had to-day and the constant pounding at it is bound to bring results.

Both the medical and dental professions do much charitable work. But too much of this will hamper the movement which needs financial support to be used to greater effect in education rather than in filling the teeth of the poor. A number of clinics have been swamped by the work which has been presented. We have such a great work be fore us that we should wisely husband our resources and put our efforts where they will mean the most. We have been teaching and the people have been learning the financial worth of sanitary mouth conditions. The Panama Canal would have been impossible without sanitary science. From a financial standpoint it is impossible to over-estimate what sanitary science has done for the country at large and what the army dental work has done to emphasize the value of dental services to the public. Our public schools are wasting a great deal of money which might be saved by proper sanitary conditions.

Dr. Proctor K. Brown, Milton—I think a careful consideration of free medical dispensary service will convince us that we ought to stop and turn back from this free dental hospital business before it is too late. It will be a great deal better for the members of this society. Already this has taken a great deal of money from the dentists. At one time there were no hospitals and free dispensaries. In the State of Massachusetts the laboring classes are generally earning large wages and should pay for their dental work. The average net profits of dentists throughout the State are not large and we are not called upon to make this sacrifice. I consider this work will damage me from $300 to $400. Many of the dentists among us are not wealthy, and work taken away from them lessens the opportunity for social and vacation enjoyment. Where a clinic is started many who could pay will take advantage of us to go there. I suppose if you gentlemen want these clinics they will come. But I do not think it fair to start anything that will work to the financial injury of the rank and file of the dental profession.

Dr. H. C. Meriam, Salem—Look at a child who cannot chew or masticate his food. Into that life we are working to bring a larger opportunity. I remember reading in a paper years ago that the loss of health and thought from bad teeth was incalculable. We must be our brother's keeper to help or destroy. I remember Dr. Eliot stating that you would be astonished to see what a dirty mouth might be found behind a fair face. This question does not deal entirely with the poor. An analysis of the air in a sweat shop, a tenement house, and a Pullman train showed that the Pullman train had the worst. It has been my practice before my patients passed from my care to instruct them about these matters. With some of the poorer people I would quickly cut away some of the decayed surface of the molars and prevent decay by the use of silver nitrate. It is astonishing how long teeth treated this way remain useful. For some years I have been telling my patients of some of the uses of sodium perborate. Now I tell them to keep their mouths clean for the sake of their children also.

Dr. Belyea—There may be some danger of injuring the financial condition of dentists by this hospital work unless it is carefully safeguarded; yet nothing that the profession has ever done has helped the reputation of the profession as this has. There is nothing that will help dentistry so much as an advertisement of this sort.

Dr. James H. Daly—I hope we shall see next year how far this little candle throws its beams. A well-known Chelsea teacher said that he could prove that there were ten thousand people in that town who had never had a bath. A gentleman stated that there were thirty-five thousand in the northwestern part of this city. The chances are ten to one that many of these homes have no bath tubs, or that they are four flights up and are filled up with coal. This goes to show how much is to be done for sanitation. I trust you will get your hospitals.

Dr. Belyea—There are safeguards in Lynn so that those who ought not to get free work will not get it. The financial condition of all the patients will be looked into to avoid complaint of this sort.

Dr. Hosley—I have considered Dr. Brown's complaint and I want to say that the dental profession of Massachusetts can well afford to give $50,000 to advertise dentistry. I believe that dentists are losing money because they are not supporting the movement for the education of the people. The more the public is educated about the value of teeth from an economic standpoint the more work there will be for the dental profession.

Dr. Flanagan—Consider the tremendous interest that has been started in dentistry by this movement. No more than five years ago it was a common thing to have physicians thinking that there was no such thing as real worth in oral prophylaxis in dentistry. These things have come in the last five years so that we have approached the time when dentistry can be considered something other than a repairing of structural injuries. We have inculcated in our patient an idea of the value of the teeth. A few years ago surgical operators learned to put masks on their own mouths, etc., so that they would not blow their breath into the cut; now they are also putting the mouth of the patient in condition so as to prevent infection. I can name a hospital in Massachusetts where the dentist is called upon to remove calcalus from the mouth of a patient about to undergo an operation. But how much better for us to call attention to the fact that we belong to a calling that is humane and that is doing all it can to improve the condition of the teeth of all the people. The medical profession has been given a great deal of recognition for writing prescriptions and doing charitable work. Now the people of Springfield are beginning to ask how much the dentists are doing for charity outside of their offices. One might as well say to Dr. Jones, you are writing me a prescription for glasses; how much are you giving the poor in the way of glasses or spectacles? The dentist may take an hour to fill one or two teeth, while the physician can see a great many patients in that time and without having to undergo the expense of materials, etc.

Now as to the question of losing practice. We dentists have inculcated the minds of the people with the idea that we simply repair teeth. It is time to set a new standard and also to stop recommending nostrums. If we knew bacteriology we could demonstrate what we know. We can destroy a certain amount of infection by the use of mouth washes and that is all.

Dr. Lindstrom—In closing this discussion I have very little to add. I might say something about the work the various local societies are doing along the line of oral hygiene. The committee has been accused of being narrow. In starting this work one has to go slow. Since this committee has been in existence, and this has been a short time only, Dr. Kirk has stated in an editorial of the Cosmos that nothing in the last twenty-five years has done so much to advance dentistry as the meeting held under its auspices a few months ago. We have done a whole lot of work. No doubt the committee of the Massachusetts will be glad to join the National Committee as soon as it has done any work. But I think it would be wrong to appoint a new committee and ignore the work done by the existing one. In Lynn every school child is receiving a copy of a pamphlet furnished by the Lynn Hygienic Council. Yet another feature which we are establishing is to get our secretary, who is a Radcliffe College girl and who has been two years in the School of Social Workers in Boston, to enter all the class rooms in Lynn and speak ten minutes on Oral Hygiene. We have found that the tooth brush is unknown to most of the children who come in. Those who have tooth brushes often share them with five or six others.—*The Journal.*

